# Establishment of an Amino Acid Nutrition Prediction Model for Laying Hens During the Brooding and Early-Growing Period

**DOI:** 10.3390/ani15213178

**Published:** 2025-10-31

**Authors:** Jiatong Li, Meng Hou, Weidong Yuan, Xin Zhang, Xing Wu, Yijie Li, Ruirui Jiang, Donghua Li, Yujie Guo, Xiangtao Kang, Yujie Gong, Yongcai Wang, Yadong Tian

**Affiliations:** 1 College of Animal Science and Technology, Henan Agricultural University, Zhengzhou 450046, China; 18836947816@163.com (J.L.); hou_meng1999@163.com (M.H.); m15937512402@163.com (W.Y.); 13783482886@163.com (X.Z.); wuxing6871@163.com (X.W.); 18749420706@163.com (Y.L.); jrrcaas@163.com (R.J.); lidonghua6656@126.com (D.L.); 15093351679@163.com (Y.G.); xtkang2001@263.net (X.K.); gongyj1118@163.com (Y.G.); 2 Henan Key Laboratory for Innovation and Utilization of Chicken Germplasm Resources, Zhengzhou 450046, China

**Keywords:** laying hen, nutrition prediction model, early-growing, amino acid, brooding

## Abstract

**Simple Summary:**

This study advances the field of poultry nutrition by introducing a dynamic modeling approach to estimate amino acid requirements for layer chicks, addressing limitations in traditional static models. This approach aligns with recent trends advocating for precision nutrition in poultry farming, where tailored feeding strategies optimize both growth efficiency and cost-effectiveness. Positioned within the literature, this research enhances the toolkit for precision livestock nutrition, offering a scalable framework applicable to other poultry species or phases. Its emphasis on adaptability and biological relevance addresses critical challenges identified in earlier studies, such as the oversimplification of nutrient requirements in heterogeneous populations.

**Abstract:**

The aim of this study was to develop a dynamic factorial model for predicting amino acid requirements in Hy-Line Gray laying hens during critical early growth stages (0–84 days), addressing the need for precision feeding in modern poultry production systems. Methods: Four sequential trials were conducted. In Trial 1, growth curves and protein deposition equations were developed based on fortnightly body composition analyses, with parameters evaluated using the Akaike and Bayesian information criteria (AIC and BIC). In Trial 2, the carcass and feather amino acid profiles were characterized via HPLC. And established the amino acid composition patterns of chicken feather protein and carcass protein (AAF and AAC). In Trial 3, maintenance requirements were quantified through nitrogen balance studies, and in Trial 4, amino acid patterns of feather protein (APD) and apparent protein digestibility (ADD) were established using an endogenous indicator method. These datasets were integrated through factorial modeling to predict age-specific nutrient demands. Results: The developed model revealed the following quantitative requirements (g/day) for 18 amino acids across developmental stages: aspartic acid (0.1–0.863), glutamic acid (0.170–1.503), serine (0.143–0.806), arginine (0.165–0.891), glycine (0.258–1.279), threonine (0.095–0.507), proline (0.253–1.207), alanine (0.131–0.718), valine (0.144–0.737), methionine (0.023–0.124), cysteine (0.102–0.682), isoleucine (0.086–0.458), leucine (0.209–1.067), phenylalanine (0.086–0.464), histidine (0.024–0.133), lysine (0.080–0.462), tyrosine (0.050–0.283), and tryptophan (0.011–0.060). The model demonstrated strong predictive validity throughout the 12-week growth period. Conclusion: This integrative approach yielded the first dynamic requirement model for Hy-Line Gray layers during early development. The factorial framework enables precise adjustment of amino acid provisions to match changing physiological needs and has high potential value in optimizing feed efficiency and supporting sustainable layer production practices.

## 1. Introduction

In livestock production, efficient performance depends on balancing nutritional supply with animal demand. Research has shown that precise amino acid provision is essential for the growth, health, and production performance of laying hens [1]. Amino acid requirements in laying hens are influenced by factors such as environmental conditions, genetic traits, production performance, and nutritional variables. For example, changes in ambient temperature can affect the metabolic energy and protein needs of laying hens, while different breeds may have distinct amino acid requirements. These complexities make predicting the digestibility of laying hens challenging [2].

The nutrition prediction model accurately estimates the nutrient requirements of laying hens, including amino acids, at various stages by integrating key parameters such as age, body weight (BW), digestibility, and egg weight. By combining existing nutritional knowledge with big data analysis, this model offers a scientific foundation to optimize feed formulation. Adjusting feed compositions to align with the dynamic nutritional needs of laying hens over time enables more precise dietary management, ultimately enhancing their health and productivity [3].

Digestibility is an important parameter for establishing amino acid nutrition prediction model. In previous studies [4], researchers typically measured terminal ileum digestibility in laying hens to represent their digestibility parameters at specific stages. This method has the advantage of avoiding the effects of microbial fermentation in the large intestine, thereby providing a more accurate reflection of amino acid digestion in feed [5]. However, as the physiological conditions of laying hens change with age, this method does not adequately capture the dynamic nature of nutrition prediction models or the variations in digestibility across different periods. So, in this study, we established a prediction model for protein and amino acid digestibility and further explored the digestibility characteristics of laying hens.

A variety of animal growth models are currently available, each designed for specific species and applications. The Von Bertalanffy growth equation is particularly suitable for livestock such as pigs, horses, cattle, and sheep, as well as poultry. The Gompertz growth model is widely applied in poultry (e.g., broilers and laying hens), livestock, and certain aquatic species, including fish and shrimp. The Logistic growth [6] model is extensively used for studying population dynamics and individual growth in insects [7], small mammals [8], and fish [9]. The Von Bertalanffy model is specifically employed to analyze the growth patterns of ruminants, such as goats and sheep. Mechanistic animal growth models are primarily utilized for pigs and other livestock to evaluate growth potential and feed conversion efficiency [10].

The factorial method is an analytical approach for analyzing nutritional requirements. It is widely adopted due to its adaptability and accuracy. This approach decomposes the complex issue of nutritional requirements into distinct components, which are systematically integrated. It enables a detailed analysis of various factors affecting amino acid needs, such as environmental conditions, genetic traits, and production performance. This comprehensive method enhances the accuracy and practicality of nutritional models. In conclusion, the amino acid nutrition prediction model developed using the factorial method provides valuable insights for both research and practical applications [11,12].

Hy-Line Gray laying hens, a commercial breed developed by Hy-Line International, are renowned for their superior egg-laying performance. However, factors such as the laying period, feeding environment, and temperature fluctuations can induce stress, resulting in oxidative damage. The nutritional requirements of laying hens vary significantly based on their breed, developmental stage, and production goals [13]. The developmental stages of laying hens are categorized into the brooding and growing period. The brooding period lasts from hatching to six weeks of age, while the growing period extends from seven weeks to the onset of egg production. The growing period is further divided into the early-growing phase (7–12 weeks) and the late-growing phase (13–16 weeks). Nutritional requirements differ markedly across these stages. During the brooding period, rapid growth necessitates substantial energy support. In contrast, the growing period is characterized by slower growth and a corresponding decrease in energy demand. These distinctions underscore the importance of precisely understanding and meeting the nutritional needs of laying hens at each stage to optimize their development and productivity [14]. This study aims to develop a dynamic prediction model for the nutritional requirements of commercial laying hens during the brooding and pre-lay periods. Using growth modeling and factorial analysis, the model seeks to enhance production performance and serve as a practical tool for commercial applications. Furthermore, it provides a foundation for the development of a more comprehensive growth prediction model for laying hens.

## 2. Materials and Methods

### 2.1. Ethics Approval

The animal study was conducted in 21 February 2024 at Lankao Xiaoming Agricultural and Animal Husbandry Youth Chicken Farm. All procedures were approved by the Institutional Animal Welfare and Utilization Committee (IACUC) of the College of Animal Science and Technology, Henan Agricultural University (Approval Number: 19-0068; Approval Date: 13 February 2024).

### 2.2. Bird Trials

Trial 1: To establish the growth curve of laying hens, a total of 138 Hy-Line Gray laying hens, which were one day old (on the day of hatch), were selected from Lankao Xiaoming Agricultural and Animal Husbandry Youth Chicken Farm. The sample size of 138 hens (23 per replicate) was allocated into 6 groups (23 hens/group) using a computer-generated random number sequence. The sample size was determined based on preliminary data to ensure adequate statistical power. The hens were raised until 85 days of age under controlled environmental conditions detailed below. The feed formula is shown in Table A1. Predefined humane endpoints included a rapid decline in body condition (>15% BW loss), severe lethargy, or inability to access feed and water. Dimensions and density: Brooding period cage size 70 cm ∗ 65 cm ∗ 40 cm (height), providing ≥200 cm^2^ per chick at 50 chicks/m^2^; growing period adjusted to 110 cm ∗ 70 cm ∗ 50 cm (height), providing ≥500 cm^2^ per chick at 20 chicks/m^2^. Brooding period: Medical absorbent cotton pads (daily replacement) for 0–3 days; transitioned to 3:1 rice husk-sawdust mixture (5–8 cm thick, partial replacement every 2 days) after 4 weeks. Growing period: 2:1 crushed corn cob-rice husk mixture (initial 8–12 cm thick, full replacement every 4 weeks), maintained at 15–20% moisture and pH 6.5–7.5. No use of perches or enrichment. 0–3 days: 24 h light, 25–30 lux (2700 K warm LED lights); 4–7 days: 22 h light, 20–25 lux; 8–21 days: 18 h light, 15–20 lux; 22–84 days: 12 h light (6: 00–18: 00), 10–15 lux. Temperature: 34–35 °C (0–3 days), gradually reduced to 24–25 °C (29–84 days), diurnal fluctuation ≤ 2 °C. Humidity: 65–70% (0–2 weeks, via humidifiers), 50–60% (3–84 weeks, via ventilation or dry bedding). Ventilation: 0.5 m^3^/(h·bird) (intermittent, 10 min/h) for 0–2 weeks; 1.0 m^3^/(h·bird) (semi-continuous, 20 min/h) for 3–4 weeks; 1.5–2.0 m^3^/(h·bird) (continuous vertical negative pressure ventilation) for 5–84 weeks. Ammonia concentration 30 cm above bedding was monitored daily, strictly controlled ≤15 ppm (complying with GB/T 18407.3-2001) [15]; ventilation and bedding replacement frequency increased when concentration reached 10–15 ppm. Chicks were grouped by uniform age and BW; behavior and physiological indicators were observed daily, with husbandry conditions adjusted as needed. The hens were managed in strict accordance with the scientific feeding management guidelines, provided with ad libitum access to feed and water, and maintained under controlled environmental conditions, including temperature, humidity, lighting, and ventilation. The specific immunization plan is as follows: 1-day-old: Marek’s disease vaccine; 7-day-old: Newcastle disease infectious bronchitis (ND-IB) combined live vaccine; 14 days old: Infectious bursal disease (Gumbolo) live vaccine; 21 days of age: booster shot combined with Newcastle disease infectious bronchitis (ND-IB) live vaccine; 35 days old: Chicken pox vaccine. Group BWs were recorded at 0, 14, 28, 42, 56, 70, and 84 days of age, with the average weight of each group calculated during each measurement. Also, mortality was recorded throughout the trial. BWt was measured daily at 10:00 AM following a standardized low-stress protocol to minimize handling-related distress. Prior to handling, environmental stressors were mitigated by reducing light intensity and turning off ventilation fans to lower noise levels. A curved barrier was used to gently restrict the birds’ movement and visual field. All personnel were trained in low-stress animal handling and maintained a calm demeanor during procedures. Birds were carefully restrained from behind and placed on a digital platform scale covered with a sterile cloth to prevent slipping. The entire process, from capture to release, was completed within 20 s per bird. For chicks, additional care was taken by fully supporting the body with both hands. After measurement, each bird was returned calmly to its home pen. Prior to weighing, birds were fasted for 4 h (for birds under 8 weeks of age) or 12 h (for birds at 8 weeks and older), respectively, with water withdrawn for 2 h to ensure accurate measurements. During each experimental phase, the average BW of all hens within a cage unit was first calculated. Following this, one hen with a BW closest to the calculated average was selected. The bird was euthanized by cervical dislocation, a method acceptable under the American Veterinary Medical Association (AVMA) Guidelines for the euthanasia of animals for birds of this size when performed by trained personnel American Veterinary Medical Association (2020) https://www.avma.org/resources-tools/avma-policies/avma-guidelines-euthanasia-animals (accessed on 15 October 2024). The procedure was carried out by an experienced researcher to ensure immediate loss of consciousness and minimize distress. No sedatives or analgesics were administered prior to euthanasia, as this method, when correctly performed, is considered rapid and humane without the need for chemical restraint. The live weight was recorded immediately thereafter. Subsequently, the feathers were manually plucked from the carcass. Both the carcass and the feathers were then weighed individually to determine their respective masses. Then dissect the carcass to remove the contents of the stomach and intestines. Measure the protein content in feathers and carcass and establish independent relationships between feather protein content and live weight, and between carcass protein content and live weight.

Trial 2: This trial aimed to establish the amino acid composition patterns in feather and carcass proteins of laying hens across different ages. It was conducted as a complementary study to Trial 1, utilizing the same population and adhering to the same ethical approval (IACUC Approval Number: 19-0068). At each weighing time point in Trial 1, one hen closest to the average BW from each of the six replicate cages was selected as a subsample (*n* = 6 per time point). Following euthanasia by cervical dislocation—a method performed by trained personnel and consistent with AVMA guidelines—samples were processed under a strict protocol to preserve integrity. The entire bird was immediately frozen at −80 °C. For analysis, carcasses were partially thawed at 4 °C, feathers were removed via a 55 °C distilled water bath (45 s) and meticulously plucked, and the eviscerated carcass was dissected in a cold room (4 °C). All samples were returned to −80 °C within a 5-day processing window. The amino acid content of 18 amino acids was subsequently measured in the feather and carcass proteins to establish their composition patterns.

Trial 3: This experiment was conducted to determine the nutrient requirements of laying hens at maintenance during different physiological stages. At 6 weeks of age (35 days old) and 11 weeks of age (77 days old), 48 hens close to the average BW were selected from the flock and randomly allocated into two treatment groups: a nitrogen-free diet group and a low-nitrogen diet group. The formulas for nitrogen free and low nitrogen diets are shown in Table A2. Nitrogen balance trials were performed at room temperature of 20–25 °C. The experimental procedure was divided into two phases: a pre-trial period and a trial period. The pre-trial period lasted 3 days, during which the normal diet and the experimental diet were mixed in a 1:1 ratio and provided to the hens. This was followed by the trial period, which lasted 4 days, during which only the experimental diet was fed. Water intake was restricted to 70% of ad libitum levels, and daily feed intake was meticulously recorded To minimize contamination from feed, dust, and feather debris, a plastic shield was installed above the trays to deflect external contaminants, and perforated plastic mats were placed on the cage floors to limit the entry of larger feathers. During collection, any small feathers present in the excreta were meticulously removed by hand. Immediately after the trial period commenced, excreta were collected over the 4-day period using dung trays. To prevent ammonia volatilization, the collected excreta were treated twice daily with saturated boric acid [16]. At the end of the trial period, residual materials such as feed, feathers, and dander in the trays were removed. The remaining excreta were weighed and sampled for the determination of amino acid maintenance patterns in laying hens. During the nitrogen-free dietary period, all birds were subjected to rigorous health monitoring twice daily to ensure their welfare. The monitoring assessed mental status (including vigilance and responsiveness), feed intake, and general condition. Predefined humane endpoints were strictly enforced, requiring the immediate removal of any bird from the trial if it exhibited a significant decline in activity, severe lethargy, or a sustained reduction in feed intake. Birds meeting these criteria were promptly provided with a standard recovery diet. To maintain experimental integrity, any removed bird was replaced by another from the same replicate group with a BW closest to the current average, and the trial for that replicate was restarted. This protocol ensured the collection of reliable data while fully adhering to animal welfare principles.

Trial 4: This trial was conducted to investigate the changes in the apparent digestibility of protein and amino acids in laying hens at different growth stages. As an integral part of the overarching study, it operated under the same ethical approval (IACUC Approval Number: 19-0068) and in accordance with the ARRIVE guidelines.

At 5, 10, 25, 35, 45, 55, 70, and 85 days of age, fecal and feed samples were collected from the population described in Trial 1. At each time point, three fresh fecal samples (approximately 60 g each) were collected from the cage floors, ensuring collection was conducted with minimal disturbance to the birds. Each sample was immediately mixed with 20 mL of 0.1 mol/L dilute hydrochloric acid to prevent ammonia volatilization and stored at −80 °C. Concurrently, 500 g of the feed diet was sampled. The protein content, amino acid content, and acid-insoluble ash (AIA) content in the excreta and feed were determined. The apparent digestibility of protein and amino acids was calculated using the AIA endogenous indicator method.

### 2.3. Sample Determination Indicators and Methods

In Trial 1 and 2, the feathers and carcasses of the collected chickens were separated, and the respective weights were recorded. The crude protein content and the levels of 18 amino acids in the feathers and carcasses were subsequently analyzed. Amino acid levels were expressed as percentages of crude protein. The amino acid content was measured in dry samples and then adjusted to reflect the content in fresh samples. For the determination of the 18 amino acids, meat and feather samples were first dried in an oven (DHG-9070) at 65 °C for 24 h, then ground using a pulverizer (FW135) and mixed uniformly with a rapid mixer (XK96-A). A portion of the prepared sample was treated with hydrochloric acid under specified conditions following the national standard method (GB/T 5009.124-2003) [17]. The content of 18 amino acids in the sample was analyzed using high-performance liquid chromatography. The moisture content in the feathers and carcasses was determined according to the national standard method (GB/T 6435-2014) [18]. The procedure involved drying sea sand in a crucible at 103 ± 2 °C for 30 min, cooling it in a desiccator, and then weighing 10 g of the sample into the same crucible. The sample was mixed and dried in the oven at 103 ± 2 °C for 4 h, then placed in a desiccator to cool to room temperature before weighing. The sample was dried again for 30 min until a constant weight was achieved. The moisture content was calculated based on the first drying step, and results were rounded to one decimal place. Finally, the amino acid content was expressed as a percentage of the crude protein in the fresh sample. For the determination of nitrogen content, an appropriate amount of the crushed and mixed sample (typically 0.5–5 g for solids or an appropriate volume for liquids) was weighed and placed in a Kjeldahl flask. To this, 10–20 mL of concentrated sulfuric acid (adjusted according to sample size), 0.2–0.5 g of copper sulfate, and 10–15 g of potassium sulfate were added. The sample was heated and digested in a fume hood, starting at low heat and gradually increasing until the solution became clear, transparent, and blue-green. After cooling, the digested solution was transferred to a distillation apparatus, where sodium hydroxide (about 40%) was added to alkalinize the solution, and distillation was carried out. Ammonia generated during distillation was absorbed in a boric acid solution, which was then titrated with a standard hydrochloric acid solution (0.05–0.1 mol/L), using a methyl red-bromocresol green mixed indicator to determine the endpoint, marked by a color change from green to light red. The nitrogen content was calculated from the amount of hydrochloric acid consumed, and crude protein content was derived accordingly. In Experiment 3, nitrogen content in the feed and excreta, creatinine content in excreta, and the levels of 18 amino acids in the fecal samples of the nitrogen-free group were determined. The methods for determining nitrogen and amino acid content were identical to those used in Trial 1 and 2. For creatinine detection, 50.0 g of homogenized fecal sample from the low-nitrogen diet group was placed into a 150 mL Erlenmeyer flask and treated with 7.5% perchloric acid. The sample was centrifuged at 3000 rpm for 15 min, and the supernatant was neutralized with 2N potassium bicarbonate. After cooling for 20 min and further centrifugation to remove potassium perchlorate, the filtrate was diluted to 100 mL. It was then treated with a blood and urine creatinine kit (sarcosine oxidase method C011-2-1) and analyzed for creatinine content using an enzyme-linked immunosorbent assay (ELISA) analyzer (Multiskan FC). In Experiment 4, the protein content, AIA content, and amino acid content in the feed and fecal samples were determined. The methods for determining amino acid and protein content were consistent with those in Trial 1 and 2. The hydrochloric acid-insoluble ash content was determined following the national standard method GB/T 23742-2009 [19]. The crucible was placed in a muffle furnace at 550 °C for at least 30 min, cooled in a desiccator, and weighed. Then, 5 g of the sample was added to the crucible, carbonized, and heated in the furnace at 550 °C for 3 h. If carbonization was observed, the sample was cooled and moistened with distilled water. After evaporation in a drying oven at 103 ± 2 °C, the crucible was reheated for 1 h, cooled, and weighed again. The residue was then transferred to a beaker containing dilute hydrochloric acid and boiled for 15 min. After filtering and washing with water, the residue on the filter paper was dried in the oven at 103 ± 2 °C for 2 h, then incinerated in the muffle furnace for 1 h, cooled, and weighed.

### 2.4. Data Processing

In trials designed to establish growth models, carcass protein prediction models, and feather protein prediction models, nonlinear regression analysis was performed using SPSS 26.0 software to fit the data and determine model parameters and their optimal estimates. Then, use the Untitled64 package in Python (3.8+) to calculate the Akaike Information Criterion (AIC) and Bayesian Information Criterion (BIC) values of the fitting results. For trials focusing on the amino acid patterns of carcass and feather proteins, analysis of variance (ANOVA) was conducted using SPSS 26.0 software, with Duncan’s test employed for multiple comparisons. Results were expressed as mean ± standard error (SE), with statistical significance set at *p* < 0.05. During the development of prediction models for protein apparent digestibility and amino acid apparent digestibility, Excel was used for data processing, including calculating average values for each replicate. Trend lines were plotted, and various curve-fitting methods were compared to identify and establish the best-fitting model.

### 2.5. Model Construction

To accurately develop the amino acid nutrition model, four trials were designed and conducted. Data from Trial 1 were analyzed and compared across three growth models—Gompertz, Logistic, and Von Bertalanffy—to assess their fitting performance. The model with the best fit was selected as the optimal growth model for laying hens.

(1) Gompertz:Y=A∗exp^−b∗exp^(−k∗t)

(2) Logistic:Y=A/(1+b∗exp^−k∗t)

(3) $$Von\;Betalanffy:Y=A*{\left(1-b*exp^{-k*t}\right)}^{3}$$

where *t* represents the age of the day; BWt represents the BW of t-day-old; k is the average growth rate; A is mature weight; b is the adjustment parameter; exp is the base of the natural logarithm. We can use the function of live weight and time BW = f(t)

The data on feather and carcass protein content of laying hens, measured in Trial 2, were incorporated into various mathematical models to determine the relationship between protein content and live BW. Based on this analysis, we established the relationships between live BW, total carcass protein (CP, in grams), and total feather protein (FP, in grams) of laying hens. Additionally, the amino acid patterns of carcass protein (AAc, %) and feather protein (AAf, %) in laying hens were determined through single-factor analysis.

(4) $$Y=b_{0}+b_{1}*X$$

(5) $$Y=b_{0}+b_{1}X+b_{2}X^{2}$$

(6) Y=b0∗X^b1

(7) Y=b0∗exp^b1X

Various mathematical models were utilized to analyze the relationships between total carcass protein, total feather protein, and live BW. The optimal model was selected based on the coefficient of determination (R^2^), and the growth curve was integrated into this model. This approach established relationships between total carcass protein (CP) and live BW, expressed as CP = f(BW), and between total feather protein (FP) and live BW, expressed as FP = f(BW). The resulting carcass and feather protein models were incorporated into the growth model, facilitating the determination of carcass protein deposition over time.

(8) PRc=CPGt=f(t)

(9) PRf=FPGt=f(t)

The term ‘C’ used in the subsequent models refers to the protein maintenance coefficient, which is a crucial correlation parameter that dynamically connects the pool of metabolically active protein to the body weight of the hens. The protein maintenance coefficient C_6–7_ of laying hens at 6–7 weeks of age, the amino acid maintenance mode AAm_6–7_ at 6–7 weeks of age, the protein maintenance coefficient C_11–12_ at 11–12 weeks of age, and the amino acid maintenance mode AAm_11–12_ at 11–12 weeks of age were obtained by trial 3. According to the protein maintenance requirement (PRm) of laying hens is PRm = C ∗ BW^0.75^, and the amino acid maintenance requirement of laying hens is AARm = C ∗ BW^0.75^ ∗ AAm. Combined with the amino acid nutrition prediction model for growth, we can obtain the amino acid maintenance requirement model of laying hens at 0–6 weeks and 7–12 weeks.

(10) $$Week_{0–6}:AARm_{0–6}=C_{0–6}*BWt*AAm_{0–6}$$

(11) $$Week_{7–12}:AARm_{7–12}=C_{7–12}*BWt*AAm_{7–12}$$

The trends in apparent protein digestibility (APD) and apparent amino acid digestibility (AAD) in laying hens were analyzed using Excel (2021) software. The curve model with the highest degree of fit was selected as the predictive model for both APD and AAD [20].

(12) $$APD=100*[1-({AIA}_{feed}/{AIA}_{feces})*({Protein}_{feed}/{Protein}_{feces})]$$

(13) $$ADD=100*[1-({AIA}_{feed}/{AIA}_{feces})*({Amino\;acids}_{feed}/{Amino\;acids}_{feces})]$$

The total amino acid requirements of laying hens at different stages can be expressed as:

(14) AAR=C∗BW^0.75∗AAmAAD+PRc∗AAcAPD+PRf∗AAfAPD

## 3. Results

### 3.1. Selection and Fitting of Growth Models

**Figure 1:**
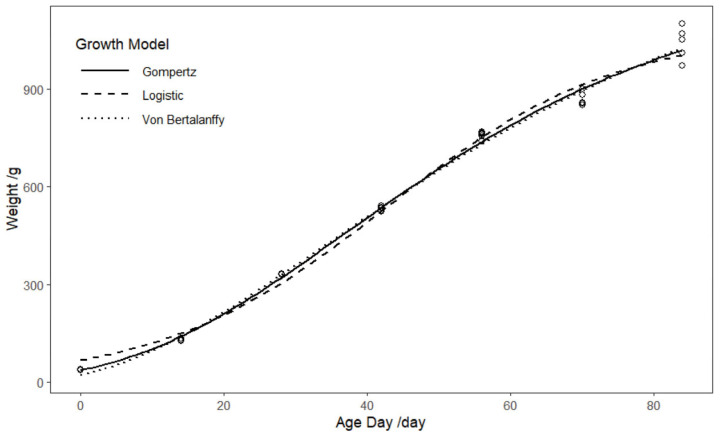
Comparison of fitting curves of three growth models.

Figure 1 shows the fitting trends of the three growth models. Table 1 shows that the biological parameters (A, b, k) for all three models are statistically significant. Among them, the Von Bertalanffy model exhibits the best fit, with an R^2^ of 0.996. Furthermore, the AIC and BIC values for the Von Bertalanffy model are lower than those for the other two models. Regarding the prediction of mature *BW*, the Von Bertalanffy model forecasts the highest weight within the 95% confidence interval, ranging from 1293.033 to 1531.803 g. The Gompertz model predicts a mature *BW* range of 1171.874 to 1323.410 g, while the Logistic model predicts the lowest mature weight, ranging from 1028.760 to 1123.305 g. The standard error for the Von Bertalanffy model’s predicted mature *BW* is relatively high, at 59.023 g. The Gompertz model predicts the inflection point at approximately 37 days of age, with an inflection *BW* of 464.183 g. The Logistic model predicts the inflection point at around 42 days, with an inflection *BW* of 395.891 g. The Von Bertalanffy model predicts the inflection point at approximately 33 days, with an inflection *BW* of 418.494 g. Based on these findings, we selected the Von Bertalanffy model as the growth prediction model for laying hens.

The model expression for determining the live weight and time of Hy-Line gray laying hens is:

(15) Von Bertalanffy:Wt=1412.418∗(1−0.748∗exp(−0.024∗t))^3

### 3.2. Establishment of Models for Total Carcass Protein, Total Feather Protein, and Live Weight, Respectively

Table A3 delineates distinct growth patterns in Hy-Line Gray laying hens across various developmental stages. During the initial phase from 0 to 14 days of age, the hens exhibited rapid growth, which persisted at a high rate from 14 to 28 days, albeit with a slight deceleration. The growth rate further moderated after 28 days but experienced a resurgence between 70 and 84 days. Concurrently, the percentage of carcass weight relative to live weight demonstrated a gradual decline as the hens matured. This decline was particularly pronounced from 0 to 42 days, after which the rate of decrease attenuated. In contrast, the percentage of feather weight relative to live weight exhibited a progressive increase, consistent with the typical developmental trajectory of laying hens. The change in feather weight percentage was negligible from 0 to 14 days but became more pronounced from 14 to 42 days, followed by a slight reduction between 42 and 56 days.

Carcass protein concentration maintained stability throughout the trial period (mean 20.95% ± 0.43%), showing only marginal depletion with advancing age. In contrast, feather protein exhibited a distinct age-dependent degradation pattern, with particularly pronounced depletion occurring between 70 and 84 days of age. Total body protein mass demonstrated significant accumulation with chronological development, though the 56–70 days interval showed limited progression. Concurrently, cumulative feather protein displayed attenuated accrual rates, manifesting marked deceleration during the 42–56 days phase, a phenomenon temporally aligned with the proportional decline of feather mass relative to live *BW*.

Using the body composition data of Hy-Line Gray laying hens at different ages (as shown in Table A3), four mathematical models were applied to analyze the relationships between carcass and feather protein content and live *BW*. The results of these analyses are presented in Table 2 and Table 3. For carcass protein, the R^2^ value of the model Y = b_0_ ∗ X^^b1^ was greater than or equal to those of the other three models, with its SE, AIC, and BIC values being smaller than those of the other models. Regarding the feather protein, the R^2^ value of the model Y = b_0_ + b_1_X + b_2_X^2^ is larger than those of the other three models, with its AIC values being smaller than those of the other three models.

Therefore, the relationships between the carcass protein, feather protein of laying hens and the live weight are determined as follows:

(16) CP=0.181∗LBW^1.007

(17) FP=−3.393+0.089LBW−(1.46∗10−5)LBW^2

By substituting the growth model (15) into Equations (16) and (17), and subsequently differentiating each formula, functional relationships were established to describe the deposition rates of carcass protein and feather protein over time.

PRc = CPGt = 14.587 ∗ ((1 − 0.748·exp(−kt))3) 1.007 ∗ exp(−0.024 ∗ t) ∗

(1/1−0.0748 ∗ exp(−0.024 ∗ t))

PRf = CPGt = −130.717 ∗ 0.024 ∗ (1 − 0.748 ∗ exp(−0.024 ∗ t)) 5 ∗ exp(−0.024 ∗ t)

+ 282.082 ∗ 0.024 ∗ (1 − 0.748·exp(−0.024·t)) 2 ∗ exp(−0.024·t)

Among them, PRc is the amount of carcass protein deposition at t-day age, and PRf is the amount of feather protein deposition at t-day age.

### 3.3. Establishment of Amino Acid Composition Patterns of Carcass Protein and Feather Protein in Laying Hens

The results of the one-way ANOVA (Table 4) revealed the amino acid composition of the carcass protein as follows: For Aspartic acid (Asp), the content of Aspartic acid showed relatively minor differences from 14 to 70 days of age (*p* > 0.05). Regarding Glutamic acid (Glu), no significant differences were found in the Glutamic acid content among the four age groups of 0–28 days and 56 days (*p* > 0.05), nor were there significant differences between 28 and 56 days (*p* > 0.05). For Serine (Ser), there were no obvious differences at 14, 42, 56, and 84 days of age (*p* > 0.05). In the case of Arginine (Arg), no significant differences were observed from 14 to 84 days of age (*p* > 0.05), and there were also no significant differences among 0, 14, 42, and 84 days of age (*p* > 0.05). For Glycine (Gly), no differences were shown from 14 to 70 days of age (*p* > 0.05), and no significant differences were presented from 28 to 84 days of age (*p* > 0.05). Except for 70 days of age, all other ages showed significant differences compared to 0 days of age (*p* < 0.05). Regarding Threonine (Thr), there were no differences at 14, 28, 42, 56, and 84 days of age (*p* > 0.05), and no significant differences were found between 0 and 14 days of age (*p* > 0.05). Compared to other ages, 0 days and 70 days of age showed significant differences (*p* < 0.05). For Proline (Pro), there were no significant differences from 14 to 56 days and 84 days of age (*p* > 0.05), while significant differences were shown at 0 and 70 days of age (*p* < 0.05). In Alanine (Ala), no significant differences were presented among 0, 14, and 70 days of age (*p* > 0.05), and no significant differences were found from 28 to 56 days of age (*p* > 0.05), and there were no significant differences between 42 and 84 days of age. Regarding Valine (Val), no significant differences were found between 28 and 56 days and 84 days of age (*p* > 0.05), and 0 and 14 days of age showed significant differences compared to other groups (*p* < 0.05). For Methionine (Met), no significant differences were observed from 0 to 70 days of age (*p* > 0.05). Compared to 0 and 42 days of age, 84 days of age showed no significant differences (*p* > 0.05), but significant differences compared to other groups (*p* < 0.05). For Cystine (Cys), no significant differences were found from 28 to 56 days of age (*p* > 0.05), and 0 days of age showed significant differences compared to other ages (*p* < 0.05). Regarding Iso Leucine (Ile), no significant differences were found between 0 and 28 days and 84 days of age (*p* > 0.05), and there were no significant differences among 0, 28, 42, and 84 days of age (*p* > 0.05), and no significant differences among 0, 42, and 70 days of age (*p* > 0.05), and no significant differences among 14, 42, and 84 days of age (*p* > 0.05). For Leucine (Leu), no significant differences were observed from 14 to 70 days of age (*p* > 0.05), while 0 and 84 days of age showed significant differences (*p* < 0.05). Regarding Phenyl Alanine (Phe), no significant differences were found from 28 to 84 days of age (*p* > 0.05), and 0 and 14 days of age showed significant differences compared to other groups (*p* < 0.05). In Histidine (His), no significant differences were found at 14, 28, and 84 days of age (*p* > 0.05), and no significant differences were found between 28 and 70 days and 0 days of age (*p* > 0.05). Regarding Lysine (Lys), no significant differences were found from 14 to 84 days of age (*p* > 0.05), and no significant differences were found between 0 and 28 days and 56 days of age (*p* > 0.05). For Tyrosine (Tyr), no significant differences were found from 42 to 84 days of age (*p* > 0.05), and no significant differences were found among 14, 28, 56, and 84 days of age (*p* > 0.05). 0 days of age showed significant differences compared to other groups. For Tryptophan (Trp), no significant differences were found among 28, 42, 70, and 84 days of age (*p* > 0.05), and no significant differences were found between 14 and 56 days of age (*p* > 0.05). 0 days of age showed significant differences compared to other groups (*p* < 0.05).

The results of the one-way ANOVA (Table 5) revealed the amino acid composition of the feather protein as follows: For Asp, no significant differences were found from 14 to 84 days of age (*p* > 0.05), and 0 days of age showed the most significant differences compared to other ages (*p* < 0.05). Regarding Glu, no significant differences were found at 14, 42, and 56 days of age (*p* > 0.05), and no significant differences were found from 28 to 84 days of age (*p* > 0.05). 0 days of age showed the most significant differences compared to other ages (*p* < 0.05). For Ser, no significant differences were found at 28, 42, and 84 days of age (*p* > 0.05), and no significant differences were found between 28 and 70 days of age (*p* > 0.05), and no significant differences were found among 0, 28, 56, and 70 days of age (*p* > 0.05), and no significant differences were found among 0, 14, and 56 days of age (*p* > 0.05). Regarding Arg, no significant differences were found from 28 to 56 days of age (*p* > 0.05), and no significant differences were found at 14, 28, and 84 days of age (*p* > 0.05), and no significant differences were found among 28, 56, and 84 days of age (*p* > 0.05). 0 days of age showed significant differences compared to other ages (*p* < 0.05). For Gly, no differences were shown between 0 and 70 days of age (*p* > 0.05), and no significant differences were presented among 0, 14, and 84 days of age (*p* > 0.05), and no significant differences were found between 14 and 28 days of age (*p* > 0.05), and no significant differences were found from 14 to 42 days of age (*p* > 0.05). Regarding Thr, no significant differences were found at 28, 42, 70, and 84 days of age (*p* > 0.05), and no significant differences were found from 14 to 42 days and 84 days of age (*p* > 0.05), and no significant differences were found among 14, 28, and 56 days of age (*p* > 0.05). 0 days of age showed significant differences compared to other ages (*p* < 0.05). For Pro, no significant differences were found from 56 to 84 days of age (*p* > 0.05), and no significant differences were found between 28 and 56 days, 0 and 14 days, and 28 and 42 days (*p* > 0.05). In Ala, no significant differences were presented from 28 to 84 days of age (*p* > 0.05), and 0 and 14 days of age showed significant differences compared to other groups, respectively (*p* < 0.05). Regarding Val, no significant differences were found between 56 and 84 days, 28 and 56 days, 14 and 42 days, and 0 and 14 days (*p* > 0.05), and 70 days of age showed no significant differences compared to other groups (*p* < 0.05). For Met, no significant differences were found from 14 to 42 days of age (*p* > 0.05), and no significant differences were found from 56 to 84 days of age (*p* > 0.05). 0 days of age showed significant differences compared to other groups (*p* < 0.05). For Cys, no significant differences were found between 28 and 42 days, 28 and 84 days, 56 and 70 days, and 0 and 14 days (*p* > 0.05). Regarding Ile, no significant differences were found from 56 to 84 days and 28 days (components) (*p* > 0.05), and no significant differences were found among 0, 14, and 56 days of age (*p* > 0.05). 14 days of age showed significant differences compared to other groups (*p* < 0.05). For Leu, no significant differences were found between 0 and 14 days of age (*p* > 0.05), and no significant differences were found between 42 and 56 days of age (*p* > 0.05), and no significant differences were found from 28 days and 56–84 days of age (*p* > 0.05). Regarding Phe, no significant differences were found from 56 to 84 days and 28 days of age (*p* > 0.05), and no significant differences were found among 14, 28, and 56 days of age (*p* > 0.05), and no significant differences were found between 14 and 56 days of age (*p* > 0.05). 0 days of age showed no significant differences compared to other ages. In His, no significant differences were found from 28 to 84 days of age (*p* > 0.05). 0 and 14 days of age showed significant differences compared to other groups, respectively (*p* < 0.05). Regarding Lys, significant differences were found from 14 to 42 days of age (*p* > 0.05), and significant differences were found from 56 to 84 days of age (*p* < 0.05). 0 days of age showed significant differences compared to other ages (*p* < 0.05). For Tyr, no significant differences were found at 28 and 42 days of age (*p* > 0.05), and no significant differences were found from 56 to 84 days of age (*p* > 0.05). 0 and 14 days of age showed significant differences compared to other ages, respectively (*p* < 0.05). Regarding Trp, no significant differences were found from 42 to 84 days of age (*p* > 0.05). 0, 14, and 28 days of age showed significant differences compared to other ages, respectively (*p* < 0.05).

The analysis revealed that the amino acid composition of carcass and feather proteins in laying hens exhibited significant dynamic changes over time. For carcass protein, most amino acid contents at 0 days of age were significantly different from those at other time points (*p* < 0.05). Between 28 and 56 days of age, the amino acid content remained relatively stable with minimal changes. Except for significant differences in Glu, Ile, Tyr, and Trp at various time points, the content of other amino acids showed no significant differences across the three time points (*p* > 0.05). Therefore, the average amino acid content observed at 28, 42, and 56 days of age can be used as a representative amino acid composition pattern for carcass protein in laying hens. For feather protein, amino acid content changes were particularly pronounced during 0–28 days of age. Most amino acid levels in feather protein at 0 days were significantly different from those at other time points (*p* < 0.05). By 56 days of age, these differences began to diminish. Between 56 and 84 days of age, the levels of Ser, Gly, Arg, Thr, Val, and Cys continued to fluctuate, while other amino acids stabilized, showing no significant differences (*p* > 0.05). Based on these findings, the average amino acid content observed at 56, 70, and 84 days of age can serve as a representative amino acid composition model for feather protein in laying hens.

It can be seen from Table A4 that:

AAc (%): Asp 6.308, Glu 11.058, Ser 2.703, Arg 5.02, Gly 7.104,Thr 3.673, Pro 4.771, Ala 5.172, Val 3.642, Met 1.732, Cys 0.775, Ile 3.179, Leu 5.486, Phe 3.108, His 1.963, Lys 5.416, Tyr 2.183, Trp 0.634.

AAf (%): Asp 5.616, Glu 9.485, Ser 8.057, Arg 5.877, Gly 6.849, Thr 4.101, Pro 9.681, Ala 4.036, Val 6.897, Met 0.438, Cys 7.943, Ile 4.085, Leu 7.516, Phe 4.276, His 0.469, Lys 1.691, Tyr 2.411, Trp 0.436.

### 3.4. Nitrogen Balance Test and Calculation of Protein Maintenance Requirement Coefficient for Laying Hens

The nitrogen balance experiment results for Hy-Line Gray laying hens are summarized in Table 6.

The endogenous nitrogen excretions during the 6th and 11th weeks of feeding a nitrogen-free diet were 217.567 mg/day and 239.645 mg/day, respectively. Under maintenance conditions, nitrogen losses from feathers, dander, and other skin coverings in the low-nitrogen diet were 160.658 mg/day and 377.823 mg/day, respectively, while creatinine excretions were 1.535 mg/day and 2.124 mg/day, respectively. The coefficient of protein maintenance requirement for the brooding period (0–6 weeks) is represented by the value at 6–7 weeks of age, while that for the early growing period (7–12 weeks) is represented by the value at 11–12 weeks of age. Accordingly, the coefficients of protein maintenance requirements for Hy-Line Gray laying hens during 0–6 weeks and 7–12 weeks are:

$$\mathrm{Week}\;0–6:\;C\;=\;\frac{\left(217.567+160.658\right)*6.25}{{(519.509+523.273)/2}^{0.75}}=21.665$$

$$\mathrm{Week}\;7–12:\;C=\frac{(239.645+377.823)*6.25}{{(885.757+867.936)/2}^{0.75}}=23.950$$

The net protein maintenance requirement of laying hens aged 0–6 weeks is

(18) $$PRm(mg/gBW^{0.75})=21.665*BW^{0.75}$$

The net protein maintenance requirement of laying hens aged 7–12 weeks is

(19) $$PRm(mg/gBW^{0.75})=23.950*BW^{0.75}$$

### 3.5. Egg Laying Chicken Amino Acid Maintenance Mode

As can be seen from Table 7 and Table 8, there was a significant decrease in the amount of nitrogen excreted in the form of amino acids, which accounted for 1/7 of the total nitrogen in laying hens at 6–7 weeks of age, and in the form of amino acids, which accounted for 1/9 of the total nitrogen in laying hens at 11–12 weeks of age. In contrast, the amount of nitrogen lost from feather dander was significantly higher in laying hens at 11–12 weeks of age, while the amount of nitrogen lost in non-amino acid and creatinine forms did not change much. We used the amino acid maintenance pattern at 6–7 weeks of age to represent the amino acid maintenance pattern during the brooding period (0–6 weeks) and the amino acid maintenance pattern at 11–12 weeks of age to represent the amino acid maintenance pattern during the early-growing period (7–12 weeks). Based on the endogenous nitrogen excretion, body surface nitrogen loss, amino acid and creatinine excretion, and amino acid composition patterns of carcass and feather proteins of Hyland’s Gray laying hens, the amino acid composition patterns were calculated for 0–6 weeks (Table 7) and 11–12 weeks (Table 8), and were noted as AAm (0–6) and AAm (7–12), respectively.

Amino acid maintenance pattern Aam (0–6) (%): Asp 6.549, Glu 11.091 Ser 5.771, Arg 5.685, Gly 9.696, Thr 4.307, Pro 7.793, Ala 5.208, Val 5.657, Met 1.199, Cys 4.311, Ile 4.049, Leu 6.974, Phe 4.051, His 1.248, Lys 3.855, Tyr 2.800, Trp 0.644.

Amino acid maintenance pattern Aam (7–12) (%): Asp 6.282, Glu 10.627, Ser 6.632, Arg 5.790, Gly 9.103, Thr 4.253, Pro 8.530, Ala 4.859, Val 6.124, Met 0.955, Cys 5.576, Ile 4.214, Leu 7.216, Phe 4.169, His 0.992, Lys 3.154, Tyr 2.750, Trp 0.589.

### 3.6. Establishment of Prediction Models for Protein and Amino Acid Apparent Digestibility in Laying Hens

**Figure 2:**
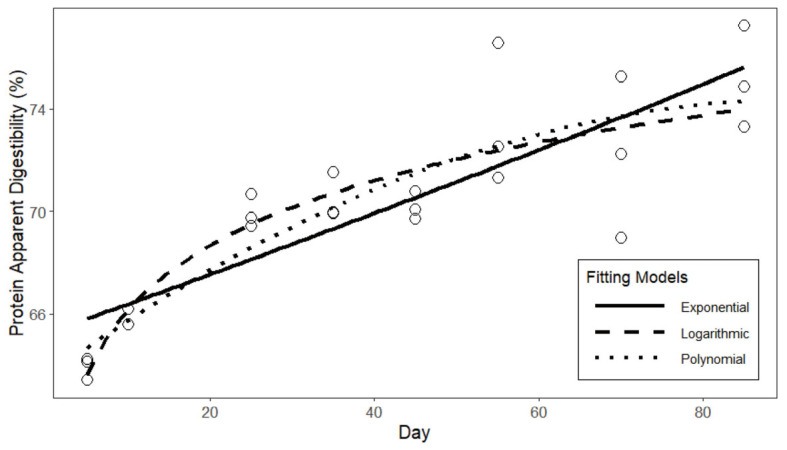
Curve fitting of protein digestibility in laying hens.

**Figure 3:**
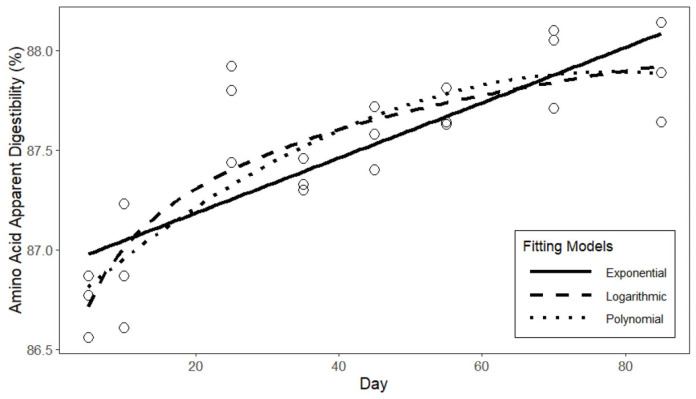
Curve fitting of amino acid digestibility in laying hens.

The fitted curves of the three models of apparent protein digestibility are presented in Figure 2. Based on the fitting results Table 9, the logarithmic function exhibits the highest degree of fit for the apparent protein digestibility of laying hens, with an R^2^ of 0.806. In comparison to the quadratic and exponential functions, the logarithmic function demonstrates a smaller standard error in the fitting results, as well as lower AIC and BIC values. In conclusion, the fitting results of the logarithmic function can be employed as the predictive model for the apparent protein digestibility of laying hens. The fitted curves of the three models of apparent amino acid digestibility are presented in Figure 3. Based on the fitting results in Table 10, similar to the protein digestibility results, the logarithmic function exhibits a higher degree of fit and smaller errors compared to the other two models. Therefore, the fitting results of the logarithmic function are selected as the predictive model for the apparent amino acid digestibility of laying hens.

(20) $$\begin{array}{l} APD=0.577+0.037*ln(t) \end{array}$$

(21) ADD=0.860+0.004∗ln(t)

### 3.7. Dynamic Model of Amino Acid Nutrition

We can assume that layers of different ages and *BW* are only accumulated in quantity, and the amino acid pattern that constitutes the protein does not change. The amino acid requirement for growth can be estimated by the quality and quantity of the deposited protein.

According to the 13th equation.

We can obtain the nutritional prediction model of amino acid requirement of laying hens at different ages.

AAR=Ct∗[1412.418∗(1−0.748∗exp−0.024∗t)3]^0.75∗AAmt0.0860+0.004∗ln(t)/1000+(14,587∗(1−0.748∗exp−0.024∗t)3)1.007∗exp−0.024∗t∗(1/1−0.0748∗exp−0.024∗t))∗AAc)0.577+0.037∗ln(t)+[−130.717∗0.024∗(1−0.748∗exp−0.024∗t)5exp−0.024∗t+282.082∗0.024∗(1−0.3748∗exp−0.024∗t)2∗exp−0.024∗t]∗AAf0.577+0.037∗ln(t)

t is the age of the hen in days. AARt is the total amino acid requirement. Ct is the maintenance coefficient, representing the correlation between BW and metabolically active protein across different growth phases. Its value is phase-specific. AAmt is the amino acid maintenance pattern for a specific growth phase. AAc is the amino acid pattern in the carcass. AAf is the amino acid pattern in feathers. The model dynamically integrates the requirements for maintenance, body tissue growth, and feather development to predict the total daily amino acid needs.

## 4. Discussion

In traditional feeding practices, producers often provide nutrients in excess of animals’ actual requirements to ensure optimal growth performance. However, this approach leads to wasted feed resources, increased production costs, and heightened environmental pollution [21,22].

Growth models, such as the Gompertz and Logistic models, are widely used in poultry farming to analyze and predict growth patterns. These models provide valuable insights into poultry development over time, support accurate growth forecasting, and assist in breeding management, thereby optimizing production performance [23]. In Trial 1, we compared three growth models and identified the Von Bertalanffy model as the most suitable for describing the growth of Hy-Line Gray laying hens. The Von Bertalanffy model is expressed as: Wt = 1412.418 ∗ (1 − 0.748 ∗ exp(−0.024 ∗ t))^3^. By calculating the model parameters, the Von Bertalanffy model predicted the mature weight of Hy-Line Gray laying hens to be 1412.418 g, with an inflection point at 33 days of age and an inflection point weight of 418.494 g. According to the 2024 Hy-Line Gray Laying Hens Feeding and Management Manual, the average BW of commercial Hy-Line Gray laying hens is 1450 g at the onset of laying (18 weeks) and reaches 1980 g by 70 weeks of age. This discrepancy can be primarily attributed to the limited observation period in this study, which concluded at 84 days of age, whereas laying hens generally attain their mature body weight at a later stage. Furthermore, as noted in the study by Kühleitner, Manfred et al. [6] the Von Bertalanffy model tends to overestimate mature weight, while the Logistic model often underestimates it. Thus, the inherent limitations of the model may also introduce certain biases in predicting body weight at advanced ages. In terms of statistical indicators, the AIC, BIC, and R^2^ values of the Gompertz and Von Bertalanffy models were quite similar. However, the mature weight predicted by the Gompertz model was 1261 g, which is even lower than the reference value provided in the management guide. Therefore, under the current data constraints, the Von Bertalanffy model was selected as the growth curve fitting tool and still offers reasonable reference value. The Von Bertalanffy, Gompertz, and Logistic growth models [24] each offer distinct advantages and disadvantages in predicting animal growth. The Von Bertalanffy model is widely utilized in biological growth studies and is applicable to a broad range of organisms, including fish and poultry. It provides the best fit during the early and middle stages of growth. However, its sensitivity to initial parameters is a notable drawback; inappropriate parameter selection can lead to poor fit results. While the Von Bertalanffy model accurately describes the growth of laying hens in this study, further investigation is needed to assess the specific impact of parameter variations on the fit. The Gompertz model has demonstrated high fitting precision in numerous studies [25] and is effective in characterizing the growth curve of poultry. It is one of the most frequently used models in poultry growth research, with many studies advocating for its application. Despite the clear biological significance of its parameters, there exists a negative correlation between the maturation rate (k) and mature BW (A), which may result in an underestimation of mature BW. The Logistic model is simple in structure [26], with fewer parameters, making it easy to understand and apply. It often provides a good fit for growth data, particularly during the early and middle stages. However, the inflection point of the Logistic model is fixed at half of the carrying capacity, which may not be suitable in some cases. In certain situations, the Logistic model may not achieve the same level of accuracy as other models, particularly in the later stages of growth.

Through Trial 2, significant differences were identified in the amino acid compositions of carcass and feather proteins in laying hens. Animal growth is primarily reflected in changes to the absolute quantities of body components, with notable increases in the absolute amounts of body protein and body fat [27]. Feather protein contains the sulfur-containing amino acids Cys and Met, with Cys levels significantly exceeding those in the carcass. This difference arises because keratin, which constitutes 89–97% of feathers, relies on Cys as its principal component. Conversely, the Met content in feather protein is lower than in the carcass. This is because most amino acids in the carcass are allocated to muscle growth and repair, whereas feather protein primarily supports feather growth and maintenance. Clearly, Cys plays a more critical role in feather development compared to Me [28].

Studies have demonstrated that Ser and Glu acid levels in feathers are significantly higher than those in carcasses, whereas Cys, Met, and Lys levels in carcasses exceed those in feathers. These findings differ from the results of the present study [29]. Carcass protein contains a relatively higher proportion of essential amino acids, with Lys being a notable example. Lys plays a crucial role in the growth and maintenance of muscle tissue, which is consistent with the findings of this study. Previous research on the amino acid requirements of laying hens was based on two key assumptions: (1) the amino acid requirement is primarily composed of two components—maintenance and growth; and (2) in laying hens of different ages and weights, protein deposition increases quantitatively while the amino acid composition of the protein remains constant. Hypothesis 1 has been widely validated, and Hypothesis 2 is also generally supported by other scholars [30].

Some researchers [29] suggest that the amino acid composition of the carcass in laying hens remains relatively stable after six weeks of age. Similarly, the feather protein composition becomes stable after three weeks of age, except for Val, Met, Lys, and Try. Based on the results of one-way ANOVA in this study, the average amino acid content at 28, 42, and 56 days of age was used as the representative model for the carcass amino acid composition of laying hens, while the amino acid content of feathers at 56, 70, and 84 days of age was used as the representative model.

Maintenance is defined as the physiological state in which the weight of healthy animals remains constant without any productive activities, and all nutrients in the body are in equilibrium. However, even in this state, metabolic processes within the body continue, resulting in the excretion of nitrogen-containing substances. Therefore, dietary protein supplementation is required to compensate for these losses. [31] In Trial 3, the BWs of chickens in the low-nitrogen groups at 6–7 weeks and 11–12 weeks of age exhibited only minor fluctuations. These slight weight changes are attributed to amino acid losses. It is hypothesized that these variations are primarily caused by changes in water intake and excretion rather than significant alterations in tissue mass [32].

The loss of nitrogen can be summarized into six items [33]: nitrogen loss from the body surface occurs due to the shedding of skin coverings. Ineffective urinary nitrogen loss results from reactive protein turnover. Additional nitrogen losses arise from the shedding of intestinal mucosal cells, bile secretion, and the release of digestive enzymes. Further losses are associated with the synthesis of non-egg nitrogenous substances, such as those related to muscle intoxication. Irreversible chemical modifications of amino acids, such as the conversion of Lys to hydroxylysine, also contribute to nitrogen loss. Finally, free amino acids are excreted in urine, further adding to the nitrogen loss.

Some scholars [34], while studying the amino acid requirements of meat ducks [35] and Taihe silky fowls, once regarded the amino acid composition in fecal and urinary excretions as that of urinary nitrogen. However, the amino acids in fecal and urinary excretions are mainly derived from the secretions of the digestive tract, as well as the sloughed mucosa and epithelium [36].

At the beginning of the 20th. century, some scholars regarded the amino acid composition of carcass protein as the amino acid composition lost in the form of urinary nitrogen, and believed that the results obtained in this way were closer to the actual situation. Liu et al. (2024) [29] used the same method to establish the amino acid nutrition maintenance model of laying hens with Jing se 6 chicks as trial materials. Compared with the results of this trial, except for glycine, Threonine and Cys, the other results were not significantly different. The variation in results may have arisen from substantial differences among various breeds of Hy-Line Gray layers in terms of body weight, daily weight gain, and production performance. These disparities contribute to variations in the requirements and proportions of certain amino acids used for maintenance and weight gain [3].

For laying hens, it changes with age. Providing appropriate nutrition at different growth stages can promote the growth and development of organs, thereby improving their performance during laying [37]. Dietary proteins are digested to release free amino acids, which are absorbed into the blood and used by various tissues to synthesize new proteins. The lack of protein can lead to slow growth and even weight loss of chickens, which seriously affects production performance. Birds of different types or ages [38] have different nutritional structures at different physiological stages, such as laying hens. As the growth stage of laying hens, growing hens are the key period of organ development and body growth, which affects the stability of egg quality during laying period. Chicks exhibit strong metabolic activity and rapid growth and development. To meet their nutritional and environmental requirements, appropriate feeding and management practices are essential. During this period, the absorption of the yolk sac in the chicks’ abdomen provides initial nutrition, but a diet rich in digestible protein is also necessary. The growing phase of hens, spanning from 7 to 18 weeks of age, is critical as it bridges the early development and laying periods. Proper feeding and management during this stage significantly influence growth, gonadal development, and, consequently, the future production performance of laying hens. Therefore, laying hens should have their corresponding nutritional requirements [39] and maintenance needs at different growth stages [40]. However, the nutritional value of feed formulations now, and there is no refinement of the nutritional needs of each period, and there is a lack of relevant data [41]. In this study, reserve laying hens aged 0 to 12 weeks were divided into two distinct stages for the nitrogen balance test. An amino acid nutrition prediction model, tailored to the developmental stages of laying hens, was subsequently established. This model facilitates the optimization of feeding strategies and provides valuable data to support the further refinement of nutrition prediction models for laying hens. The digestive system [42] of newly hatched laying hens is underdeveloped. As hens age, their digestive organs mature progressively, leading to a continuous increase in the secretion and activity of digestive enzymes. This, in turn, improves the digestibility of proteins and amino acids. For instance, during the initial weeks after hatching, chicks exhibit a limited ability to digest proteins and amino acids. However, this capacity gradually improves during the growing and laying periods, aligning with the findings of this study. Over the years, there have been relatively few reports on the application of curve models to predict the variations in the [43] digestibility of laying hens. The digestibility of laying hens is influenced by multiple factors. Developing a prediction model for their digestibility aids in accurately forecasting variations in digestibility and assessing related factors such as feed composition, feeding environment, and age. In this study, Hy-Line Gray laying hens aged 0–12 weeks were used to establish relationships between APD, ADD, and age [44]. Compared to the widely adopted napparent amino acid digestibility (NPU) and standardized ileal digestibility of amino acids (SID AA), apparent digestibility values may be relatively higher because the effects of intestinal microbial fermentation are not excluded. Nevertheless, the prediction trend strongly correlates with observed changes in the digestibility of laying hens. Thus, these findings hold referential significance and provide a foundation for developing a comprehensive digestibility prediction model for laying hens.

As presented in Table 11, the daily amino acid requirements (g/day) for laying hens across age stages (0 to 84 days), modeled via R-based analysis, demonstrated significant deviations from established nutritional benchmarks. Met requirements (0.023–0.124 g/day) remained below recommended levels at all ages, while Thr (0.095–0.507 g/day) and Val (0.144–0.737 g/day) aligned closely with standard recommendations. Notably, Glu and Gly exhibited pronounced overestimations, with modeled values escalating from 0.170 g/day to 1.503 g/day Glu and 0.258 g/day to 1.147 g/day Gly, substantially exceeding established thresholds. These discrepancies suggest potential model calibration issues, particularly in accounting for endogenous synthesis of non-essential amino acids or dynamic phase-specific nutrient partitioning. The modeled Met requirements across all age stages were consistently below recommended levels (Met 253 mg/d) [3,45] potentially attributable to its specialized role in protein synthesis and interconversion between sulfur-containing amino acids. Notably, while this study did not account for endogenous metabolic pathways, the combined Met + Cys values exceeded recommendations from 42 days onward, with significant elevations during 56–84 days, aligning closely with production-phase demands. Thr and Val requirements closely matched established benchmarks [46], underscoring their criticality in maintaining physiological homeostasis and growth performance. Conversely, Glu and Gly requirements substantially surpassed recommendations, likely due to their classification as non-essential amino acids [47]; the omission of endogenous synthesis mechanisms in the model may have inflated calculated values. These findings provide a comprehensive characterization of age-specific amino acid requirements in laying hens, offering scientific guidance for formulating precision diets to optimize health and productivity.

Overall, the model predictions are in line with previous studies. Any discrep-ancies may arise from the dynamic changes in protein increase and chemical composition within poultry during the growth process, thereby influencing the model’s assessment of amino acid nutritional requirements. Additionally, the variation in the growth rates of different layer hen breeds contributes to distinct protein and amino acid requirements reflecting breed-specific characteristics. To validate the accuracy of the model, further experiments will be conducted.

## 5. Conclusions

In this study, a dynamic prediction model of protein and amino acid requirements of y-Line Gray laying hens during the brooding period was established. The model-predicted amino acid requirements for Hy-Line Gray layers from 0 to 84 days of age exhibited distinct patterns: Asp (0.1–0.863 g/day), Glu (0.170–1.503 g/day), Ser (0.143–0.806 g/day), Arg (0.165–0.891 g/day), Gly (0.258–1.279 g/day), Thr (0.095–0.507 g/day), Pro (0.253–1.207 g/day), Ala (0.131–0.718 g/day), Val (0.144–0.737 g/day), Met (0.023–0.124 g/day), Cys (0.102–0.682 g/day), Ile (0.086–0.458 g/day), Leu (0.209–1.067 g/day), Phe (0.086–0.464 g/day), His (0.024–0.133 g/day), Lys (0.080–0.462 g/day), Tyr (0.050–0.283 g/day), and Try (0.011–0.060 g/day). This model also provided an important reference for assessing the nutritional requirements of other animals, which will help improve production efficiency throughout the animal industry [48].

## Figures and Tables

**Table 1:** Fitting results of three growth models for laying hens.

Model	Gompertz	Logistic	Von Bertalanffy
Model Expression	y = 1261.652 ∗ exp(−3.521 ∗ exp(−0.034 ∗ t))	y = 1076.032/(1 + exp(14.881 + (0.063) ∗ t))	y = 1412.418 ∗ (1 − 0.748 ∗ exp(−k ∗ t))3
Predicting Mature Weight ^1^ A/g	1261.652	1076.032	1412.418
95% CI	1171.874–1323.410	1028.760–1123.305	1293.03–1531.803
Length of the CI ^2^	151.536	96.78	238.77
SE ^3^	37.427	23.371	59.022803
Correlation coefficient b ^2^	3.521	14.881	0.748
95% CI	3.247–3.761	12.141–17.620	0.711–0.785
Length of the CI	0.514	0.372	0.074
SE	0.127	1.354	0.018
Growth rate coefficient k ^3^	0.034	0.063	0.024
95% CI	0.030–0.037	0.057–0.069	0.021–0.027
Length of the CI	0.007	0.012	0.006
SE	0.002	0.003	0.001
R^2^	0.994	0.991	0.993
AIC	392.13	701.04	389.48
BIC	397.27	706.18	394.62
Inflection time/day	37.382	42.762	33.667
Weight of inflection/g	464.183	395.891	418.494

^1^ Predicted maximum weight of mature laying hens. ^2^ It quantifies uncertainty around an index estimate, not the probability of the parameter lying within a specific interval. ^3^ SE, root mean squared error; R^2^ (coefficient of determination): higher values indicate a more parsimonious model fit; AIC, Akaike information criterion; BIC, Bayesian information criterion: smaller values indicate a more parsimonious model fit.

**Table 2:** Parameters of different equations for determining the relationship between carcass protein content and live BW.

	b0 ^1^	b1 ^1^	b2 ^1^	R^2 2^	SE ^2^	AIC ^2^	BIC ^2^
Y = b_0_ + b_1_X	0.221	0.189		0.998	2.248	40.291	40.129
Y = b_0_ + b_1_X + b_2_X^2^	1.662	0.18	9.02 ∗ 10^−6^	0.998	3.202	41.540	41.323
Y = b_0_ ∗ X^^b1^	0.181	1.007		0.998	0.039	40.240	40.075
Y = b_0_ ∗ exp^^b1X^	33.423	0.002		0.943	7.645	64.810	64.648

^1^ b_0_, b_1_, and b_2_ are parameters in the model. ^2^ SE, root mean squared error; R^2^ (coefficient of determination): higher values indicate a more parsimonious model fit. AIC, Akaike information criterion; BIC, Bayesian information criterion: smaller values indicate a more parsimonious model fit.

**Table 3:** Parameters of different equations for determining the relationship between feather protein content and live BW.

	b_0_^1^	b_1_^1^	b_2_^1^	R^2 2^	SE ^2^	AIC ^2^	BIC ^2^
Y = b_0_ + b_1_X	−1.064	0.073		0.979	3.044	45.440	44.377
Y = b_0_ + b_1_X + b_2_X^2^	−3.393	0.089	−1.46 ∗ 10^−5^	0.982	4.232	44.454	45.227
Y = b_0_ ∗ X^^b1^	0.075	0.993		0.979	0.116	45.703	44.541
Y = b_0_ ∗ exp^^b1X^	12.967	0.002		0.888	4.241	56.339	56.177

^1^ b_0_, b_1_, and b_2_ are parameters in the model. ^2^ SE, root mean squared error; R^2^ (coefficient of determination): higher values indicate a more parsimonious model fit. AIC, Akaike information criterion; BIC, Bayesian information criterion: smaller values indicate a more parsimonious model fit.

**Table 4:** Amino acid pattern (% of protein) in defeathered carcasses of Hy-Line Gray laying hens.

	0 Day	14 Days	28 Days	42 Days	56 Days	70 Days	84 Days
Asp	6.485 ± 0.005 a ^1^	6.264 ± 0.027 bc	6.318 ± 0.014 bc	6.380 ± 0.028 abc	6.226 ± 0.024 c	6.318 ± 0.011 bc	6.407 ± 0.029 ab
Glu	10.923 ± 0.005 b	10.868 ± 0.046 b	11.000 ± 0.026 ab	11.193 ± 0.036 a	10.982 ± 0.051 ab	10.590 ± 0.017 c	11.217 ± 0.042 c
Ser	3.120 ± 0.024 a	2.64 ± 0.014 c	2.860 ± 0.015 b	2.665 ± 0.031 c	2.584 ± 0.011 c	2.322 ± 0.003 d	2.722 ± 0.026 bc
Arg	5.155 ± 0.009 a	5.08 ± 0.020 ab	4.987 ± 0.010 b	5.067 ± 0.015 ab	5.008 ± 0.022 b	4.998 ± 0.014 b	5.095 ± 0.012 ab
Gly	6.640 ± 0.045 c	6.858 ± 0.022 bc	7.047 ± 0.019 ab	7.138 ± 0.038 ab	7.126 ± 0.063 ab	6.900 ± 0.059 abc	7.207 ± 0.041 a
Thr	3.478 ± 0.010 b	3.57 ± 0.015 ab	3.713 ± 0.007 a	3.660 ± 0.033 a	3.646 ± 0.017 a	3.044 ± 0.004 c	3.697 ± 0.025 a
Pro	4.963 ± 0.029 a	4.752 ± 0.015 b	4.740 ± 0.013 b	4.783 ± 0.019 b	4.790 ± 0.021 b	4.440 ± 0.028 c	4.807 ± 0.023 b
Ala	4.992 ± 0.011 c	5.044 ± 0.019 c	5.143 ± 0.010 b	5.213 ± 0.009 ab	5.160 ± 0.024 b	4.972 ± 0.012 c	5.277 ± 0.010 a
Val	4.070 ± 0.011 a	3.798 ± 0.021 b	3.620 ± 0.010 cd	3.637 ± 0.013 c	3.668 ± 0.015 c	3.526 ± 0.012 d	3.590 ± 0.018 cd
Met	1.750 ± 0.003 ab	1.712 ± 0.009 b	1.725 ± 0.007 b	1.758 ± 0.012 ab	1.714 ± 0.013 b	1.700 ± 0.007 b	1.803 ± 0.013 a
Cys	1.217 ± 0.025 a	0.878 ± 0.007 b	0.765 ± 0.007 cd	0.745 ± 0.010 cd	0.814 ± 0.010 bc	0.700 ± 0.007 d	0.712 ± 0.007 d
Ile	3.213 ± 0.032 abc	3.106 ± 0.017 cd	3.197 ± 0.015 abc	3.295 ± 0.015 ab	3.044 ± 0.020 d	3.336 ± 0.010 a	3.163 ± 0.016 bcd
Leu	5.840 ± 0.013 a	5.542 ± 0.024 b	5.482 ± 0.017 b	5.543 ± 0.024 b	5.432 ± 0.015 b	5.488 ± 0.019 b	5.510 ± 0.021 b
Phe	3.527 ± 0.005 a	3.212 ± 0.014 b	3.100 ± 0.010 c	3.115 ± 0.011 c	3.108 ± 0.010 c	3.074 ± 0.015 c	3.107 ± 0.014 c
His	1.933 ± 0.026 b	2.152 ± 0.015 a	2.020 ± 0.013 ab	1.960 ± 0.032 b	1.910 ± 0.013 b	1.918 ± 0.011 b	2.025 ± 0.033 ab
Lys	5.198 ± 0.018 b	5.398 ± 0.035 ab	5.390 ± 0.029 ab	5.442 ± 0.030 a	5.416 ± 0.036 ab	5.508 ± 0.028 a	5.578 ± 0.033 a
Tyr	2.490 ± 0.015 a	2.262 ± 0.013 b	2.258 ± 0.011 b	2.092 ± 0.022 c	2.215 ± 0.014 bc	2.132 ± 0.010 c	2.152 ± 0.017 bc
Try	0.748 ± 0.005 a	0.676 ± 0.004 b	0.598 ± 0.005 c	0.617 ± 0.004 c	0.688 ± 0.006 b	0.606 ± 0.003 c	0.595 ± 0.007 c

^1^ The data in the table are $\bar{X}\pm SE$. The comparison between the seven-day-old data from the same line reveals that the difference marked with the same letter is not significant (*p* > 0.05), and the difference marked with different letters is significant (*p* < 0.05). The sample size *n* = 6, and the degrees of freedom df = 6 − 2 = 4.

**Table 5:** Amino acid pattern of protein (% of protein) in feathers of Hy-Line Gray laying hens.

	0 Day	14 Days	28 Days	42 Days	56 Days	70 Days	84 Days
Asp	6.002 ± 0.083 a ^1^	5.672 ± 0.018 bc	5.798 ± 0.013 b	5.558 ± 0.041 c	5.528 ± 0.031 c	5.665 ± 0.016 bc	5.655 ± 0.022 bc
Glu	8.318 ± 0.034 c	9.120 ± 0.034 b	9.553 ± 0.033 a	9.275 ± 0.071 ab	9.293 ± 0.051 ab	9.578 ± 0.030 a	9.583 ± 0.030 a
Ser	7.792 ± 0.060 cd	7.418 ± 0.051 d	8.080 ± 0.059 abc	8.522 ± 0.090 a	7.793 ± 0.055 cd	8.00 ± 0.049 bc	8.378 ± 0.050 ab
Arg	6.990 ± 0.045 a	5.900 ± 0.010 c	5.688 ± 0.011 cde	5.525 ± 0.048 e	5.647 ± 0.033 de	6.130 ± 0.020 b	5.855 ± 0.015 cd
Gly	6.875 ± 0.045 ab	6.658 ± 0.019 bcd	6.618 ± 0.015 cd	6.563 ± 0.040 d	6.638 ± 0.035 cd	7.067 ± 0.009 a	6.843 ± 0.025 bc
Thr	3.683 ± 0.007 d	4.017 ± 0.009 bc	4.115 ± 0.009 abc	4.157 ± 0.037 ab	3.988 ± 0.021 c	4.172 ± 0.016 a	4.143 ± 0.013 ab
Pro	8.613 ± 0.047 d	8.57 ± 0.0110 d	9.368 ± 0.025 bc	9.105 ± 0.082 c	9.488 ± 0.065 ab	9.733 ± 0.039 a	9.822 ± 0.032 a
Ala	2.882 ± 0.015 c	3.743 ± 0.011 b	4.037 ± 0.012 a	3.943 ± 0.025 a	3.957 ± 0.027 a	4.082 ± 0.005 a	4.070 ± 0.022 a
Val	5.732 ± 0.040 e	5.980 ± 0.008 de	6.542 ± 0.023 c	6.157 ± 0.062 d	6.643 ± 0.054 bc	7.167 ± 0.027 a	6.882 ± 0.022 b
Met	0.322 ± 0.003 c	0.493 ± 0.004 a	0.480 ± 0.003 a	0.493 ± 0.004 a	0.437 ± 0.003 b	0.443 ± 0.004 b	0.435 ± 0.003 b
Cys	7.177 ± 0.035 e	7.140 ± 0.049 e	8.653 ± 0.065 ab	8.855 ± 0.104 a	7.920 ± 0.049 cd	7.632 ± 0.048 d	8.277 ± 0.061 bc
Ile	3.890 ± 0.028 bc	3.607 ± 0.01 d	4.007 ± 0.013 abc	3.830 ± 0.045 c	4.010 ± 0.031 abc	4.055 ± 0.012 ab	4.190 ± 0.015 a
Leu	6.875 ± 0.033 c	6.820 ± 0.007 c	7.517 ± 0.018 a	7.142 ± 0.060 b	7.377 ± 0.051 ab	7.562 ± 0.024 a	7.610 ± 0.026 a
Phe	5.093 ± 0.034 a	4.103 ± 0.007 cd	4.243 ± 0.007 bc	4.070 ± 0.035 d	4.183 ± 0.026 bcd	4.310 ± 0.015 b	4.335 ± 0.014 b
His	1.967 ± 0.041 a	0.847 ± 0.009 b	0.513 ± 0.009 c	0.458 ± 0.004 c	0.477 ± 0.007 c	0.457 ± 0.005 c	0.473 ± 0.007 c
Lys	1.503 ± 0.018 c	2.020 ± 0.024 a	1.930 ± 0.023 a	1.905 ± 0.013 a	1.697 ± 0.015 b	1.693 ± 0.022 b	1.683 ± 0.018 b
Tyr	4.260 ± 0.023 a	2.892 ± 0.017 b	2.632 ± 0.010 c	2.583 ± 0.024 c	2.402 ± 0.016 d	2.382 ± 0.016 d	2.448 ± 0.013 d
Try	0.617 ± 0.007 a	0.565 ± 0.005 b	0.498 ± 0.002 c	0.467 ± 0.004 d	0.433 ± 0.006 d	0.433 ± 0.002 d	0.440 ± 0.003 d

^1^ The data in the table are $\bar{X}\pm SE$. The comparison between the seven-day-old data from the same line reveals that the difference marked with the same letter is not significant (*p* > 0.05), and the difference marked with different letters is significant (*p* < 0.05). The sample size *n* = 6, and the degrees of freedom df = 6 − 2 = 4.

**Table 6:** Results of nitrogen balance trials in Hy-Line Gray laying hens.

	Week 6–7		Week 11–12	
Item	Low-Nitrogen Group	Non-Nitrogen Group	Low-Nitrogen Group	Non-Nitrogen Group
Initial body weight, g	519.509 ± 18.419 ^1^	487.82 ± 8.759	867.936 ± 6.983	873.221 ± 8.283
Final body weight, g	523.273 ± 17.022	421.379 ± 6.387	885.757 ± 12.839	840.339 ± 5.563
Feed intake, g/day	40.627 ± 2.200	20.961 ± 0.893	240.315 ± 5.779	161.983 ± 12.156
N intake, mg/day	520.136 ± 26.545	18.781 ± 0.799	724.789 ± 17.431	36.284 ± 2.722
N excretion, mg/day	359.478 ± 4.986	217.567 ± 8.486	346.966 ± 14.300	239.645 ± 19.991
N retention, mg/day	160.658 ± 8.717		377.823 ± 18.502	
Creatinine excretion, mg/day	1.535 ± 0.061		2.124 ± 0.050	

^1^ The data in the table are $\bar{X}\pm SE$. The sample size is *n* = 6, and the degrees of freedom df = 6 − 2 = 4.

**Table 7:** Amino acid maintenance patterns and loss in laying hens aged 6–7 weeks.

Item	Endogenous Amino Acid Loss			Loss of Feather Dander ^3^ (mg/day)	Total Loss (mg/day)	Amino Acid Maintenance Mode ^4^ (%)
	Amino Acid Pattern ^1^ (mg/day)	Non-Amino Acid Pattern ^2^ (mg/day)	Creatinine Pattern ^1^ (mg/day)			
Nitrogen	53.085 ± 5.068	163.912 ± 0.023	0.570 ± 0.007	160.658 ± 8.717	378.225	%
Asp	30.792 ± 2.815	64.755 ± 0.340		59.282 ± 2.034	154.829	6.549
Glu	48.518 ± 4.660	113.564 ± 0.582		100.119 ± 3.537	262.201	11.091
Ser	23.706 ± 2.596	27.711 ± 0.824		85.005 ± 2.778	136.422	5.771
Arg	18.396 ± 1.748	51.543 ± 0.241	2.364 ± 0.095	62.099 ± 3.242	134.402	5.685
Gly	82.767 ± 7.940	73.071 ± 0.492	1.019 ± 0.041	72.350 ± 3.352	229.207	9.696
Thr	20.805 ± 2.131	37.720 ± 0.163		43.297 ± 1.71	101.822	4.307
Pro	33.035 ± 3.718	48.996 ± 0.251		102.179 ± 3.414	184.21	7.793
Ala	27.364 ± 2.585	53.138 ± 0.227		42.610 ± 1.531	123.112	5.208
Val	23.470 ± 2.298	37.382 ± 0.216		72.869 ± 3.660	133.721	5.657
Met	3.875 ± 0.383	17.819 ± 0.100	2.025 ± 0.081	4.627 ± 0.161	28.346	1.199
Cys	10.282 ± 1.344	7.916 ± 0.190		83.718 ± 1.297	101.916	4.311
Ile	20.083 ± 2.916	32.550 ± 0.761		43.099 ± 1.219	95.732	4.049
Leu	29.266 ± 2.867	56.276 ± 0.267		79.330 ± 2.654	164.872	6.974
Phe	18.751 ± 1.669	31.888 ± 0.069		45.134 ± 1.568	95.773	4.051
His	4.371 ± 0.399	20.175 ± 0.276		4.945 ± 0.079	29.491	1.248
Lys	17.613 ± 1.916	55.669 ± 0.240		17.849 ± 0.538	91.131	3.855
Tyr	18.332 ± 1.88	22.418 ± 0.510		25.430 ± 0.603	66.18	2.800
Try	4.102 ± 0.408	6.533 ± 0.314		4.593 ± 0.123	15.228	0.644

^1^ Obtained through the collection of excreta through testing experiments. ^2^ The nitrogen loss in nonamino acid form is obtained by subtracting the nitrogen loss in amino acid form and creatinine form from the total nitrogen loss measured by the nitrogen-free feed group, calculating the crude protein content by nitrogen loss and multiplying it by AAC to obtain the loss of various amino acids. ^3^ The amount of nitrogen lost from feather dander was estimated by measuring the nitrogen retention in the low-nitrogen feed group and then the amount of crude protein lost was calculated. Multiplying the total amount of crude protein by AAf yielded the amount of various amino acids lost. ^4^ The percentages of various amino acids lost to crude protein content were calculated by calculating the total protein content lost through the loss of total nitrogen.

**Table 8:** Maintenance of amino acid patterns and shunts in laying hens aged 11–12 weeks.

Item	Endogenous Amino Acid Loss			Loss of Feather Dander ^3^ (mg/day)	Total Loss (mg/day)	Amino Acid Maintenance ^4^ Mode (%)
	Amino Acid Pattern ^1^ (mg/day)	Non-Amino Acid Pattern ^2^ (mg/day)	Creatinine Pattern ^1^ (mg/day)			
Nitrogen	70.351 ± 8.047	168.502 ± 8.040	0.789 ± 0.018	377.823 ± 18.502	617.465	%
Asp	39.233 ± 4.115	66.569 ± 0.350		136.646 ± 4.689	242.448	6.282
Glu	62.601 ± 7.336	116.744 ± 0.598		230.779 ± 8.154	410.124	10.627
Ser	31.53 ± 3.653	28.487 ± 0.847		195.941 ± 6.403	255.958	6.632
Arg	24.064 ± 2.797	52.987 ± 0.247	3.271 ± 0.078	143.14 ± 7.472	223.462	5.790
Gly	107.997 ± 10.135	75.117 ± 0.505	1.409 ± 0.034	166.769 ± 7.727	351.292	9.103
Thr	27.567 ± 3.232	38.777 ± 0.168		99.800 ± 3.942	164.144	4.253
Pro	43.308 ± 4.795	50.368 ± 0.258		235.525 ± 7.869	329.201	8.530
Ala	34.668 ± 3.917	54.626 ± 0.233		98.218 ± 3.529	187.512	4.859
Val	29.953 ± 2.901	38.429 ± 0.222		167.966 ± 8.436	236.345	6.124
Met	5.062 ± 0.789	18.318 ± 0.103	2.802 ± 0.067	10.666 ± 0.370	36.848	0.955
Cys	14.070 ± 1.607	8.137 ± 0.196		192.973 ± 2.989	215.18	5.576
Ile	29.829 ± 7.988	33.462 ± 0.782		99.344 ± 2.809	162.635	4.214
Leu	37.786 ± 4.275	57.852 ± 0.274		182.858 ± 6.117	278.496	7.216
Phe	24.064 ± 2.480	32.781 ± 0.071		104.035 ± 3.615	160.88	4.169
His	6.143 ± 1.154	20.74 ± 0.284		11.398 ± 0.183	38.281	0.992
Lys	23.367 ± 4.275	57.227 ± 0.247		41.143 ± 1.239	121.737	3.154
Tyr	24.448 ± 3.166	23.046 ± 0.524		58.618 ± 1.39	106.112	2.750
Try	5.410 ± 0.593	6.716 ± 0.323		10.587 ± 0.283	22.713	0.589

^1^ Obtained through the collection of excreta through testing experiments. ^2^ The nitrogen loss in nonamino acid form is obtained by subtracting the nitrogen loss in amino acid form and creatinine form from the total nitrogen loss measured by the nitrogen-free feed group, calculating the crude protein content by nitrogen loss and multiplying it by AAC to obtain the loss of various amino acids. ^3^ The amount of nitrogen lost from feather dander was estimated by measuring the nitrogen retention in the low-nitrogen feed group and then the amount of crude protein lost was calculated. Multiplying the total amount of crude protein by AAf yielded the amount of various amino acids lost. ^4^ The percentages of various amino acids lost to crude protein content were calculated by calculating the total protein content lost through the loss of total nitrogen.

**Table 9:** Regression results of the apparent protein digestibility curve.

	Exponential Function	Logarithmic Function	Polynomial Function
Model expression	y = 0.651 ∗ e^1.7710413e−3∗t^	y = 0.577 + 0.037 ∗ ln(t)	y = 0.635 + 0.0023 ∗ t − 1.25260 ∗ 10^−5^ ∗ t^2^
R^2 1^	0.748	0.806	0.791
SE	0.028	0.017	0.018
AIC	−101.215	−124.724	−120.823
BIC	−98.859	−122.368	−117.289
*p*	0.000 **	0.000 **	0.000 **
Regression coefficient_1_ *p*	0.000 **	0.000 **	0.000 **
Regression coefficient_2_ *p*			0.048 *

^1^ R^2^: (coefficient of determination): higher values indicate a more parsimonious model fit; SE, root mean squared error; AIC, Akaike information criterion; BIC, Bayesian information criterion: smaller values indicate a more parsimonious model fit. * *p* < 0.05; ** *p* < 0.01.

**Table 10:** Regression results of the apparent digestibility curve of amino acids.

	Exponential Function	Logarithmic Function	Polynomial Function
Model expression	y = 0.869 ∗ e^1.600844e−4∗t^	y = 0.860 + 0.004 ∗ ln(t)	y = 0.867 + 0.0003 ∗ day − 1.5716 ∗ 10^−6^ ∗ t^2^
R^2 1^	0.611	0.675	0.656
SE	0.004	0.003	0.003
AIC	−201.316	−212.084	−208.72
BIC	−198.96	−209.727	−205.185
*p*	0.000 **	0.000 **	0.000 **
Regression coefficient_1_ *p*	0.000 **	0.000 **	0.000 **
Regression coefficient_2_ *p*			0.116

^1^ R^2^: (coefficient of determination): higher values indicate a more parsimonious model fit; SE, root mean squared error; AIC, Akaike information criterion; BIC, Bayesian information criterion: smaller values indicate a more parsimonious model fit. ** *p* < 0.01.

**Table 11:** The requirements for various amino acids for laying hens at different ages.

Amino Acid (g/day)	0 Days	14 Days	28 Days	42 Days	56 Days	70 Days	84 Days
Asp	0.1	0.256	0.388	0.456	0.742	0.812	0.863
Glu	0.170	0.501	0.736	0.854	1.304	1.421	1.503
Ser	0.143	0.422	0.621	0.715	0.693	0.758	0.806
Arg	0.165	0.538	0.783	0.891	0.619	0.678	0.721
Gly	0.258	0.792	1.131	1.279	0.991	1.081	1.147
Thr	0.095	0.301	0.440	0.507	0.413	0.453	0.482
Pro	0.253	0.743	1.056	1.207	1.043	1.137	1.206
Ala	0.131	0.438	0.632	0.718	0.481	0.527	0.561
Val	0.144	0.446	0.646	0.737	0.629	0.688	0.731
Met	0.023	0.075	0.109	0.124	0.077	0.085	0.090
Cys	0.102	0.305	0.436	0.498	0.587	0.642	0.682
Ile	0.086	0.273	0.398	0.458	0.390	0.428	0.455
Leu	0.209	0.647	0.934	1.067	0.852	0.930	0.987
Phe	0.086	0.276	0.403	0.464	0.387	0.424	0.451
His	0.024	0.079	0.116	0.133	0.081	0.089	0.095
Lys	0.080	0.282	0.407	0.462	0.307	0.337	0.358
Tyr	0.050	0.170	0.247	0.283	0.227	0.249	0.265
Try	0.011	0.036	0.052	0.060	0.048	0.053	0.056

## Data Availability

The original contributions presented in this study are included in the article. Further inquiries can be directed to the corresponding authors.

## Abbreviations

The following abbreviations are used in this manuscript:

AIC	Akaike Information Criterio
BIC	Bayesian Information Criterion
BWt	BWt, Body weight of t-day-old
AAc	The amino acid patterns of carcass protein
AAf	Amino acid patterns of feather protein
APD	Apparent protein digestibility
ADD	Apparent amino acid digestibility

## Appendix A

**Table A1:** Composition and nutritional values of the basal diet.

Ingredient, %	Day 0–15	Day 16–85
Corn	52.11	54.07
Soybean meal	34.00	31.35
Wheat bran	2.50	3.20
Soybean oil	4.00	4.00
CaHPO_4_	0.50	0.46
CaCO_3_	2.18	2.24
Salt	0.42	0.42
Lysine	0.07	0.03
Methionine	0.17	0.17
Cystine	0.05	0.04
Threonine	-	0.02
Vitamin and mineral premix ^1^	4	4
Total	100	100
Nutrient composition ^2^		
ME, MJ/kg	12.88	12.89
Crude protein, %	19.92	19.02
Lysine, %	1.10	1.01
Methionine, %	0.46	0.45
Methionine + Cysteine, %	0.84	0.81
Threonine, %	0.75	0.73
Na, %	0.18	0.18
Cl, %	0.30	0.30
Ca, %	1.00	1.00
Available Phosphorus, %	0.46	0.45

^1^ Vitamin and mineral premix provided/kg diet: iron, 100 mg; copper, 8 mg; manganese, 20 mg; zinc, 100 mg; selenium, 0.3 mg; iodine, 0.7 mg; retinyl acetate, 10,280 IU; cholecalciferol 2280 IU; dl-a-tocopheryl acetate, 17.12 mg; menadione, 6.82 mg; thiamin, 2.28 mg; riboflavin, 5.68 mg; pantothenic acid, 12.25 mg; pyridoxine, 2.28 mg; niacin, 22.84 mg; biotin, 0.18 mg; folic acid, 1.12 mg. ^2^ Metabolic energy is calculated with reference to the “China Feed Composition and Nutrient Value Table (35th Edition)” [49]. The rest are measured values.

**Table A2:** Feed Formula for Nitrogen Balance Test.

Ingredient (%)	Low-Nitrogen Diet	Now-Nitrogen Diet
Corn starch	39	45
Glucose	38.8	44.4
Sucrose	1	1
Soybean meal	12	-
Soybean oil	2	1
Vitamin mixture ^A^	0.1	0.1
Trace mineral mixture ^B^	0.1	0.1
Choline chloride	0.2	0.2
Corn cob meal	3.8	4
Methionine	0.1	-
Limestone	0.8	0.7
Calcium hydrogen phosphate	1.8	2.0
Salt	0.3	0.3
Potassium carbonate	-	0.7
Magnesium carbonate	-	0.5
Nutrient level		
ME (MJ/kg) ^D^	12.57	12.55
CP (%) ^D^	5.37	0.37

^A^ Each kilogram of complex vitamin A contains: VA 1,000,000 IU, VD 2,500,000 IU, VK3 3000 mg, VB1 3000 mg, VB2 5000 mg, VB6 1000 mg, VB12 10 mg, niacin 34,000 mg, calcium pantothenate 12,000 mg, folic acid 500 mg, biotin 200 mg; ^B^ Each kilogram of complex trace element B contains: Fe 22,500 mg, Cu 60,000 mg, Zn 48,750 mg, Mn 75,000 mg, Se 100 mg; ^D^ is the measured value.

**Table A3:** Live weight and corresponding body composition of Hy-Line Gray laying hens at different ages.

Body Composition	Age						
	0	14	28	42	56	70	84
Live BW, g	39.240 ± 0.496	129.142 ± 0.808	329.953 ± 1.284	531.47 ± 2.942	757.44 ± 5.115	861.368 ± 8.142	1064.390 ± 21.186
Carcass weight/Live BW, %	94.726 ± 0.009	94.123 ± 0.004	93.162 ± 0.004	91.273 ± 0.004	91.872 ± 0.002	90.495 ± 0.004	90.745 ± 0.003
Feather weight/Live BW, %	5.274 ± 0.009	5.974 ± 0.004	6.726 ± 0.004	9.002 ± 0.004	7.938 ± 0.002	9.604 ± 0.004	9.222 ± 0.002
Protein concertration in carcass, %	21.501 ± 0.125	20.810 ± 0.161	21.060 ± 0.137	21.148 ± 0.111	20.703 ± 0.145	20.193 ± 0.132	21.297 ± 0.095
Protein concertration in feather, %	88.587 ± 0.267	88.623 ± 0.254	89.083 ± 0.523	89.433 ± 0.059	82.513 ± 1.866	83.133 ± 0.728	73.457 ± 1.532
Amount of carcass protein, g	7.995 ± 0.162	25.297 ± 0.189	61.662 ± 0.291	102.58 ± 0.409	144.071 ± 1.198	157.41 ± 1.860	205.674 ± 3.931
Amount of feather protein, g	1.769 ± 0.019	7.120 ± 0.400	20.719 ± 0.776	42.838 ± 1.524	50.783 ± 1.542	68.035 ± 1.852	72.465 ± 3.106

Note: The data in the table is $\bar{X}\pm SE$. The sample size *n* = 6, and the degrees of freedom df = 6 − 2 = 4.

**Table A4:** Amino acid patterns of carcass protein and feather protein in laying hens.

Amino Acid	Aspartic Acid	Glutamic Acid	Serine	Arginine	Glycine	Threonine	Proline	Alanine	Valine	Methionine	Cysteine	Isoleucine	Leucine	Phenylalanine	Histidine	Lysine	Tyrosine	Tryptophan
AAC (%)	6.308 ± 0.026	11.058 ± 0.039	2.703 ± 0.047	5.020 ± 0.014	7.104 ± 0.017	3.673 ± 0.012	4.771 ± 0.009	5.172 ± 0.012	3.642 ± 0.008	1.732 ± 0.008	0.775 ± 0.012	3.179 ± 0.042	5.486 ± 0.019	3.108 ± 0.003	1.963 ± 0.018	5.416 ± 0.009	2.183 ± 0.028	0.634 ± 0.016
AAf (%)	5.616 ± 0.025	9.485 ± 0.055	8.057 ± 0.099	5.877 ± 0.081	6.849 ± 0.071	4.101 ± 0.033	9.681 ± 0.058	4.036 ± 0.023	6.897 ± 0.087	0.438 ± 0.001	7.943 ± 0.108	4.085 ± 0.031	7.516 ± 0.041	4.276 ± 0.027	0.469 ± 0.004	1.691 ± 0.002	2.411 ± 0.011	0.436 ± 0.001

Note: The data in the table are $\bar{X}\pm SE$, $\bar{X}$ represents the percentage of various amino acids in crude protein. The sample size n = 3, and the degrees of freedom df = 3 − 2 = 1.
